# Supplementary material for: Modelled impact of Tiny Targets on the distribution and abundance of riverine tsetse
Source: PLoS Negl Trop Dis. 2024 Apr 16;18(4):e0011578. doi: 10.1371/journal.pntd.0011578 (PMC11051647; doi:10.1371/journal.pntd.0011578)
Supplement: S1 Fig — (DOCX) [file pntd.0011578.s001.docx]

S1 Figure


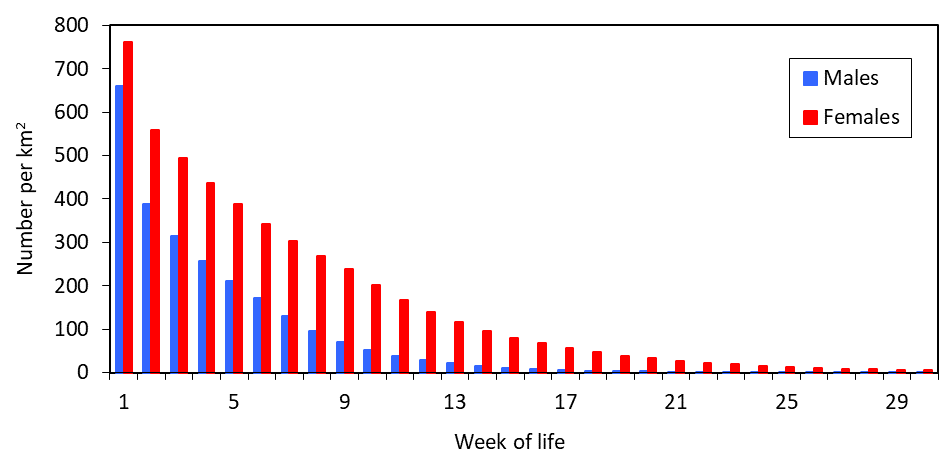


S1 Figure. Numbers of tsetse per km^2^ of habitat in various weeks of adult life, for a standard stable population of tsetse confined to the best habitat, i.e., beside a large river.
