# Supplementary material for: Modelled impact of Tiny Targets on the distribution and abundance of riverine tsetse
Source: PLoS Negl Trop Dis. 2024 Apr 16;18(4):e0011578. doi: 10.1371/journal.pntd.0011578 (PMC11051647; doi:10.1371/journal.pntd.0011578)
Supplement: S1 Table — The indicated timing of reproduction applies to tsetse in all habitats. Effects on death rates of adults and pupae in other habitats are detailed in the main text. (DOCX) [file pntd.0011578.s002.docx]

**S1** **Table.** Data for the standard stable population of tsetse confined to the best habitat, i.e., beside a large river. The indicated timing of reproduction applies to tsetse in all habitats. Effects on death rates of adults and pupae in other habitats are detailed in the main text.

| Item |  | Males | Females | Sources |
| --- | --- | --- | --- | --- |
| Population per km^2^ | Adults | 2500 | 5000 | 1 |
|  | Viable pupae | 3786 | 3516 | Model |
| Daily adult death rate | Maximum (day after emergence) | 0.142 | 0.086 | Model, 2 |
|  | Minimum (young mature adults) * | 0.028 | 0.017 | Model, 2 |
|  | Older adults ** | 0.043 | 0.026 | Model, 2 |
|  | Average for all age classes | 0.054 | 0.027 | Model, 2 |
| Pupal death rate | Per pupal period | 0.300 | 0.300 | 3 |
| Breeding | Age at dropping first larva, days | NA | 16 | 4 |
|  | Interlarval period, days | NA | 10 | 4 |
|  | Pupal duration, days | 28 | 26 | 3 |
|  | Daily production of larvae per km^2^ | 193 | 193 | Model |
|  | Daily emergence per km^2^ | 135 | 135 | Model |

* Age 10-40 days for males and 10-60 days for females.

** Age >41 days for males and >60 days for females.

1. Glasgow JP. The distribution and abundance of tsetse. Oxford: Pergamon: 1963. 214 p.

2. Hargrove, JW, Ouifki, R. & Ameh, J. E. (2011) A general model for mortality in adult tsetse (Glossina spp). Medical and Veterinary Entomology, 25, 385-394.

3. Hargrove JW, Vale GA. (2020) Models for the rates of pupal development, fat consumption and mortality in tsetse (Glossina spp). Bulletin of Entomological Research, 110, 44-56. https://doi.org/10.1017/S0007485319000233.

4. Hargrove, JW. (1994) Reproductive rates of tsetse flies in the field in Zimbabwe. Physiological Entomology 19, 307-318.
