# Supplementary material for: Modelled impact of Tiny Targets on the distribution and abundance of riverine tsetse
Source: PLoS Negl Trop Dis. 2024 Apr 16;18(4):e0011578. doi: 10.1371/journal.pntd.0011578 (PMC11051647; doi:10.1371/journal.pntd.0011578)
Supplement: S2 Table — (DOCX) [file pntd.0011578.s003.docx]

**S2 Table.** Initial stable population of male plus female tsetse per cell before any control in the five 13 km lengths of river studied in Phase 1, the average percentage of the initial population remaining in the last six months of control, and the average percent remaining in the upstream half of the 13 km length, expressed as a proportion of percent remaining in the downstream half, in a number of runs of the model involving increased or decreased values of one parameter in turn, in the context of standard values for all other parameters.

| *Parameter changed* | Initial tsetse per cell | Population remaining | |
| --- | --- | --- | --- |
| Value tested | Mean (range) | Mean% (range) | Up/down index |
| *No change. All parameters at standard values* | | |  |
| See Notes | 210 (134-319) | 29 (1-79) | 0.7198 |
| *Mobility pattern. Characterised by mean net movement of adult females per day* | | |  |
| 200m | 234 (139-384) | 35 (0-92) | 0.8221 |
| 600m | 193 (131-278) | 26 (3-72) | 0.6702 |
| *Growth potential. Ratio of death rates for standard stable density: zero density* | | |  |
| 1:0.95 | 18 (7-31) | 26 (1-81) | 0.5303 |
| 1:0.85 | 331 (241-468) | 32 (2-81) | 0.7988 |
| *Natural deaths. Ratio for large river:medium river: small river:interfluve* | | |  |
| 1:1.025:1.050, 1.100 | 309 (233-425) | 30 (2-79) | 0.8271 |
| 1:1.075:1.150, 1.300 | 125 (62-212) | 28 (1-79) | 0.5791 |
| *Habit cover in cells. Percent for large river:medium river:small river:interfluve* | | |  |
| 10:8:6:1 | 225 (136-357) | 28 (2-75) | 0.7141 |
| 10:6:2:1 | 200 (134-290) | 30 (1-82) | 0.7274 |
| *Readiness to enter interfluves. Percent of random entry* | | |  |
| 0.10% | 211 (135-322) | 29 (1-79) | 0.7186 |
| 10.00% | 193 (121-296) | 30 (2-79) | 0.7316 |
| *Schedule of daily kill rates at a target. Characterised by % kill of old females* | | |  |
| Schedule 0.01 | 210 (134-319) | 57 (22-91) | 0.8134 |
| Schedule 0.10 | As above | 24 (0-75) | 0.7219 |
| *Rate of target degradation. Percent per day* | | |  |
| 1.0% | As above | 26 (1-77) | 0.7179 |
| 2.0% | As above | 32 (3-80) | 0.726 |
| *Duration of control programme* |  |  |  |
| 6 months | As above | 47 (9-93) | 0.8658 |
| 2 years | As above | 19 (1-66) | 0.5444 |
