# Supplementary material for: Modelled impact of Tiny Targets on the distribution and abundance of riverine tsetse
Source: PLoS Negl Trop Dis. 2024 Apr 16;18(4):e0011578. doi: 10.1371/journal.pntd.0011578 (PMC11051647; doi:10.1371/journal.pntd.0011578)
Supplement: S3 Table — Figures in parentheses indicate the range of the percents remaining along the individual 1km-long sections of the treated plots. The locations of the plots are indicated in Fig 1B of the main part of the paper. (DOCX) [file pntd.0011578.s004.docx]

**S3 Table.** Simulated average percent of males plus females remaining in the last six months of control in the five 7km-long plots treated in Phase 1, when all of the standard parameters applied, except for a single change involving either the use of the low kill rates of Schedule 0.01 or 0.02, or the high degradation rates of 4.5% or 5.5% per day. Figures in parentheses indicate the range of the percents remaining along the individual 1km-long sections of the treated plots. The locations of the plots are indicated in Fig. 1B of the main part of the paper.

| Change made | Plot number and name | | | | |
| --- | --- | --- | --- | --- | --- |
|  | 2. Ayi | 3. Kubala | 4. Alivu | 6. Inve | 7. Arua |
| Schedule 0.01 | 42 (33-58) | 54 (47-66) | 65 (61-73) | 44 (34-64) | 59 (51-73) |
| Schedule 0.02 | 25 (16-41) | 34 (26-49) | 47 (41-57) | 27 (17-48) | 40 (31-58) |
| Degradation 4.5% | 28 (19-44) | 37 (29-52) | 50 (44-60) | 31 (21-52) | 44 (35-61) |
| Degradation 5.5% | 33 (25-49) | 43 (35-57) | 55 (50-64) | 36 (26-56) | 50 (41-66) |
