# Supplementary material for: Modelled impact of Tiny Targets on the distribution and abundance of riverine tsetse
Source: PLoS Negl Trop Dis. 2024 Apr 16;18(4):e0011578. doi: 10.1371/journal.pntd.0011578 (PMC11051647; doi:10.1371/journal.pntd.0011578)
Supplement: S1 Text — (DOCX) [file pntd.0011578.s005.docx]

**SI Text: Quantifying the changing abundance of tsetse along a river**

**Introduction**

To assess whether the abundance of tsetse was greater in downstream sections, we re-analysed data presented previously by Hope et al. [1].

**Methods**

We studied catches from a series of five pairs of pyramidal traps [2] deployed along the Kochi river (Fig. 1). Each trap was operated for an average of 5 days/month (range, 1-10 days) for 38 months, between September 2011 and October 2014, as part of a larger trial to quantify the impact of Tiny Targets on tsetse populations [1, 3]. Catches from the traps were collected and counted daily.


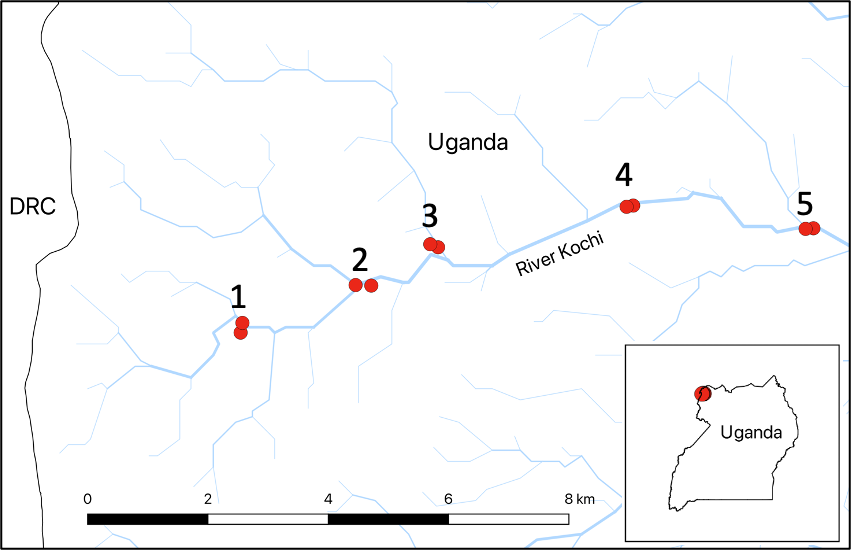


Figure 1. Location of (1-5) pairs of traps (•) along the river Kochi, Koboko district, Uganda. Base layer of rivers and national border is derived from [4].

No Tiny Targets were deployed along the river Kochi between September 2011 and October 2014. Hence, the catches provided a measure of natural variation in the abundance of tsetse in the absence of any control efforts. For present purposes, we re-analysed these data to compare catches between five pairs of traps arranged along the Kochi to test the hypothesis that catches were smaller at the more upstream sites. Relative to the most upstream pair (Pair 1), the other pairs were 2, 4, 7 and 11 km downstream.

Analyses were carried out using the open-source statistical software R [5]. We used the ‘glmmTMB’ package to fit generalized linear mixed models (glmm) to catch data using a negative binomial data distribution. To visualise the overall temporal trends of each pair of traps, site and day were specified as random effects and months as a fixed effect. Model outputs were used to estimate mean daily catches and their 95% Confidence Intervals for each month. To assess the statistical significance of differences in catch between pairs of traps, pairs was specified as a fixed effect and site and day as random effects. The statistical significance of differences were assessed using the ‘pairs’ function from the ‘emmeans’ package.

**Results**

In general, the mean daily catches were smaller in the upstream sections of the river and the trends were consistent for all months (Fig.2). For instance, mean daily catches for each month for Pair 1 varied between 0.3 tsetse/trap (0.17-0.58, 95% CI) and 1.7 (1.00-2.98) tsetse/day compared to 2.1 (1.38-3.30) – 4.9 (3.25-7.29) tsetse/trap for Pair 5. The mean catch of the most upstream pair (Pair 1) was significantly smaller than all the downstream ones (Table 1) while catches from the three downstream pairs (Pairs 3-5) were not significantly different. Pair 2 was intermediate, being significantly greater than Pair 1 and significantly smaller than Pairs 3-4.





Figure 2. Mean daily catches from trap Pairs 1-5 for each month (January-December). Inset shows the mean daily catch for each pair.

Table 1. Z-ratios for contrasts between pairs of traps.

| Pair | 1 | 2 | 3 | 4 | 5 |
| --- | --- | --- | --- | --- | --- |
| 1 |  | 4.94*** | 7.70*** | 7.70*** | 5.81*** |
| 2 |  |  | 2.78** | 2.79** | 0.91ns |
| 3 |  |  |  | 0.01ns | -1.86ns |
| 4 |  |  |  |  | -1.87ns |
| 5 |  |  |  |  |  |

***, P<0.001, **, P<0.001, ns, P>0.05

**Conclusion**

Catches of tsetse were smaller in the upstream sections of the river Kochi, consistent with the hypothesis that the abundance of tsetse is lower in upstream sections where suitable habitat is less extensive.

**References**

1. Hope A, Mugenyi A, Esterhuizen J, Tirados I, Cunningham L, Garrod G, et al. Scaling up of tsetse control to eliminate Gambian sleeping sickness in northern Uganda. PLoS Neglected Tropical Diseases. 2022;16(6). doi: 10.1371/journal.pntd.0010222. PubMed PMID: WOS:000922612200005.

2. Gouteux JP, Lancien J. The pyramidal trap for collecting and controlling tsetse flies (Diptera: Glossinidae). Comparative trials and description of new collecting techniques. . Tropical Medicine and Parasitology. 1986;37(1):61-6. PubMed PMID: WOS:A1986A785400013.

3. Tirados I, Esterhuizen J, Kovacic V, Mangwiro TNC, Vale GA, Hastings I, et al. Tsetse Control and Gambian Sleeping Sickness; Implications for Control Strategy. PLoS Neglected Tropical Diseases. 2015;9(8):e0003822. doi: 10.1371/journal.pntd.0003822.

4. Stanton MC, Esterhuizen J, Tirados I, Betts H, Torr SJ. The development of high resolution maps of tsetse abundance to guide interventions against human African trypanosomiasis in northern Uganda. Parasites & Vectors. 2018;11. doi: 10.1186/s13071-018-2922-5. PubMed PMID: WOS:000434663800003.

5. R_Core_Team, . R: A Language and Environment for Statistical Computing. In: R_Foundation_for_Statistical_Computing, editor. Vienna, Austria: <<https://www.R-project.org/>>. 2023.
