# Supplementary material for: Modelled impact of Tiny Targets on the distribution and abundance of riverine tsetse
Source: PLoS Negl Trop Dis. 2024 Apr 16;18(4):e0011578. doi: 10.1371/journal.pntd.0011578 (PMC11051647; doi:10.1371/journal.pntd.0011578)
Supplement: Attachment — Submitted filename: Mugenyi_reviewer comments.doc [file pntd.0011578.s007.doc]

**Method**

The study is about application of models in determining population dynamics of the riverine tsetse fly. Unfortunately the script does not specify any objectives for the study. These need to be stated clearly as a basis for aligning the study design and its appropriateness. There is no hypothesis stated or being tested. Hence hard to determine if the sample size is sufficient to address the hypothesis test.

**Results**

The analysis is dependent on several caveats (assumptions or limitations). These do make the model simplistic. Thus the results are based on these caveats. Under this circumstance the results are well discussed. The figures are well presented and of sufficient quality although not easy to interpret.

**Conclusions**

There is no specific section gazetted as CONCLUSION. It is hard to tell what the conclusions were made. The script instead ends with THEORETICAL CONSIDERATIONS.

**Editorial and Data Presentation Modifications?**

Minor revision recommended. This should proper allow structuring of the paper to accommodate objectives, hypothesis and proper conclusion.

**Summary and General Comments**

This work is good and very important for vector control operations and monitoring. The methodology is novel and only needs to be strengthened by including the element or factor of seasonality. Input data (i.e wet and dry season) need to be segregated.
