# Supplementary material for: Modelled impact of Tiny Targets on the distribution and abundance of riverine tsetse
Source: PLoS Negl Trop Dis. 2024 Apr 16;18(4):e0011578. doi: 10.1371/journal.pntd.0011578 (PMC11051647; doi:10.1371/journal.pntd.0011578)
Supplement: Attachment — Submitted filename: Response to Reviewers 20240318.docx [file pntd.0011578.s008.docx]

**Response to Reviewers**

| **Reviewer** |  | **Comment** |
| --- | --- | --- |
| **1** |  | Methods are well presented. |
|  | **Response** | We thank the reviewer for their compliments. |
| **1** |  | Line 124, there was some rogue highlighting on the web address. Line 134, N-S watershed may not be intuitive to an outsider, recommend updating to 'watershed running north to south' or similar Line 138, Close quarters repetition with 'of the' suggest the 2nd one changes to 'with in' Line 333. A double bracket is after [15]] |
|  | **Response** | Changes made as suggested. |
| **2** |  | The analysis is dependent on several caveats (assumptions or limitations). These do make the model simplistic. Thus the results are based on these caveats. Under this circumstance the results are well discussed. The figures are well presented and of sufficient quality although not easy to interpret. |
|  | **Response** | We thank the reviewer for their compliments. The reviewer states that the figures are ‘well presented and of sufficient quality although not easy to interpret’. The reviewer does not suggest or recommend any changes to the figures and so we have not made any. |
| **2** |  | There is no specific section gazetted as CONCLUSION. It is hard to tell what the conclusions were made. The script instead ends with THEORETICAL CONSIDERATIONS. |
|  | **Response** | We have added a final paragraph focussing on the practical conclusions from our work. |
| **2** |  | Minor revision recommended. This should proper allow structuring of the paper to accommodate objectives, hypothesis and proper conclusion. |
|  | **Response** | Thank you for the constructive recommendation. The final paragraph of the Introduction attempted to set out the three objectives of the study but the phrasing we used (“Three aspects of the field experience were of particular interest…”) was clearly inadequate. We have re-written this paragraph to make the objectives and hypotheses clearer. Our objective was to develop and then use the model to answer three research questions. We have also added practical conclusions to our theoretical considerations at the end of the manuscript. |
| **2** |  | This work is good and very important for vector control operations and monitoring. The methodology is novel and only needs to be strengthened by including the element or factor of seasonality. Input data (i.e wet and dry season) need to be segregated. |
|  | **Response** | We thank the reviewer for their compliments. We agree that the model could be improved by allowing modelling of seasonal variation in the abundance of tsetse populations and this is one of the limitations that we identified in our original draft. Adding this facility would be a major addition to the model but is outside of the scope of the present study.  In a separate paper submitted to PLOS Neglected Tropical Diseases (preprint: https://doi.org/10.1101/2023.11.21.568004) we present data showing that for *G. f. fuscipes* in NW Uganda, seasonal fluctuations in the abundance and age structure of tsetse populations are relatively modest. Hence, we believe that the model outputs and conclusions in the manuscript will not be grossly affected by not allowing for seasonal variation. We have added comments making this point in the manuscript. |
| **2** |  | The study is about application of models in determining population dynamics of the riverine tsetse fly. Unfortunately the script does not specify any objectives for the study. These need to be stated clearly as a basis for aligning the study design and its appropriateness. There is no hypothesis stated or being tested. Hence hard to determine if the sample size is sufficient to address the hypothesis test. |
|  | **Response** | Please see the response above. We have made changes to the introduction, setting out the objectives and hypotheses more clearly. We hope that this satisfies the reviewer. This is a modelling study without any empirical sampling. |
| **3** |  | Conclusions supported by the analysis and the limitations of the model are discussed. |
|  | **Response** | No changes requested or required. |
| **3** |  | Conclusions supported by the analysis and the limitations of the model are discussed. |
|  | **Response** | No changes requested or required. |
| **3** |  | Only editorial suggestion is correcting line 333 to remove extra bracket i.e [15]] |
|  | **Response** | Corrected – see response to Reviewer 1. |
| **3** |  | Congratulations on an excellent manuscript on the development of an improved model to test the impact of targets on riverine tsetse fly distribution. |
|  | **Response** | We thank the reviewer for the compliments. |
